# Contemporary Trends and Future Prospects of Genetically Engineered Vaccines in the Management and Prevention of Dental Caries

**DOI:** 10.7759/cureus.88491

**Published:** 2025-07-22

**Authors:** Sayem A Mulla, Waseem Z Khan, Laresh N Mistry, Amit H Patil, Parag Gangurde, Tanvi Shukla

**Affiliations:** 1 Dentistry, Bharati Vidyapeeth (Deemed to be University) Dental College and Hospital, Navi Mumbai, IND; 2 Orthodontics and Dentofacial Orthopaedics, Bharati Vidyapeeth (Deemed to be University) Dental College and Hospital, Navi Mumbai, IND; 3 Pedodontics and Preventive Dentistry, Bharati Vidyapeeth (Deemed to be University) Dental College and Hospital, Navi Mumbai, IND; 4 Conservative Dentistry and Endodontics, Bharati Vidyapeeth (Deemed to be University) Dental College and Hospital, Navi Mumbai, IND

**Keywords:** bcs3-l1, caries vaccine, dental caries, dental caries prevention, genetically modified vaccine, prevention of dental caries

## Abstract

Despite conventional preventive measures including dental sealants, dietary changes, and fluoride treatments, dental caries (DC) is still a global health issue. An important pathogen in DC development, *Streptococcus mutans*, forms biofilms and demineralizes enamel in acidic oral conditions. Promising substitutes are provided by new preventative techniques such as genetic engineering, probiotics, and antibacterial peptides. A potential long-term remedy is the genetically altered BCS3-L1 strain, which is intended to outcompete *S. mutans* and stop the generation of acid. Even though these developments have the potential to completely transform oral healthcare, further study is required to guarantee their efficacy, safety, and public acceptability. The aim of this review is to highlight the salient features, the probable mechanism behind it, while also exploring the limitations associated with it and shedding light on the future prospects of the strain.

## Introduction and background

The fact that dental caries (DC) is still a major worldwide health issue emphasizes the shortcomings of current preventative strategies and the intricate interactions between various factors that contribute to its development [1]. Although fluoride treatment has dramatically decreased the prevalence of dental cavities in many communities, its efficacy is sometimes hampered by uneven application, limited access to fluoridated water, and inequalities in the availability of dental care. Similar to this, dietary changes like cutting back on sugar call for long-term behavioral adjustments that can be difficult to carry out, especially in cultures where processed foods and sugar-filled drinks are common [2]. Although dental sealants offer an extra degree of security, especially for kids, their application necessitates expert assistance, which may not always be available to marginalized groups. These difficulties highlight the need to investigate alternate approaches that tackle both personal habits and more extensive structural impediments to successful caries prevention [3].

Lack of access to dental treatment increases the burden of DC in many underprivileged and disadvantaged populations, resulting in a circle of untreated oral health problems that can cause pain, infection, and a decline in general well-being. Higher rates of untreated decay and related consequences arise from people's inability to seek prompt preventative treatment due to financial limitations, a lack of awareness, and an inadequate healthcare infrastructure. Inequalities in oral health education also led to a lack of knowledge about the significance of early intervention, appropriate hygiene habits, and dietary choices [4]. The surge of DC cases worldwide in spite of preventative measures indicates that creative, affordable solutions catered to the particular requirements of these susceptible groups must be added to standard techniques.

In order to enhance oral health outcomes, emerging caries prevention strategies emphasize the use of innovative technology, community-based therapies, and microbiome-targeted techniques. New developments in probiotics, antimicrobial peptides, and customized preventive treatment present viable substitutes for lowering the burden of cariogenic bacteria and strengthening the body's defences against dental decay. Community-driven oral health initiatives, tele dentistry, and mobile dental clinics can all assist close the access gap and make sure that people who need preventative treatment can get it [5]. Furthermore, including oral health education into public health campaigns and school curriculum can promote enduring behaviours that support long-term caries prevention. These cutting-edge strategies have the potential to lessen the worldwide burden of DC and advance universal oral health by targeting the biological and socioeconomic factors of DC [6].

## Review

Methodology

A literature search was carried out on multiple scholarly databases such as PubMed, ScienceDirect, Web of Science and Google Scholar to identify scholarly articles. The keywords used included "genetically engineered vaccines", "dental caries", and "genetically modified vaccines" in various combinations of Boolean operators. Only studies available in the English language were selected, and no restriction on publication year was applied. The initial search results were then screened by authors SAM, WZK and LNM for their relevance for inclusion in this narrative article. After careful assessment, a total of 28 articles were included in this narrative synthesis.

Role of *Streptococcus mutans* in DC

DC, as we all know, is a multifactorial disease (Table 1) [7]. However, *Streptococcus mutans*, a gram-positive facultative anaerobe that is essential to the creation of plaque and the generation of acid, is the main culprit in the pathophysiology of DC. An extremely versatile and opportunistic bacterium, *S. mutans *flourishes in the oral cavity, especially when fermentable carbohydrates like sucrose, glucose, and fructose are present [8]. It produces extracellular polysaccharides, mostly glucans, which promote the formation of biofilm (dental plaque), giving it a remarkable capacity to stick to the enamel surface. Plaque development produces a confined microenvironment in which *S. mutans* may grow while evading the host's immune system and salivary purification systems. The bacteria produce organic acids, mostly lactic acid, as a result of their effective glycolysis process, which turns dietary carbohydrates into energy [9]. Because of its acidogenic properties, the biofilm's pH decreases, increasing the acidity of the oral environment. Since hydroxyapatite, the main mineral component of enamel, dissolves in low pH settings, the enamel becomes demineralized as a result of the ongoing acidic circumstances. Repeated acid assaults erode the enamel over time, eventually resulting in the development of cavities [8].

**Table 1:** Relationship between various etiological factors and dental caries.

Factor	Impact on dental caries
*Streptococcus mutans *colonization	Forms biofilm and initiates plaque development
Carbohydrate metabolism	Converts sugars into lactic acid, leading to enamel erosion
Acid production	Causes demineralization of enamel
Fluoride therapy	Increases enamel resistance but requires continuous application
Socioeconomic status	Affects access to preventive care and treatment

Apart from being acidogenic, *S. mutans *is also aciduric, which means that it can live and grow in acidic environments that would be harmful to other oral bacteria. It has a competitive edge thanks to this capability, which enables it to control the microbial population in plaque. In addition, *S. mutans* has virulence factors such as bacteriocins, which prevent the development of competing bacteria, and glucosyltransferases, which help produce sticky glucans that promote bacterial colonization. High levels of *S. mutans *are closely linked to a higher risk of DC, particularly in cases where frequent use of carbohydrates and poor oral hygiene are present. In order to prevent plaque buildup and neutralize acid production, preventive methods including consistent brushing, flossing, fluoride treatment, and dietary changes are essential [8-10]. Specifically, fluoride reduces the cariogenic potential of *S. mutans *by inhibiting bacterial metabolism and aiding in the remineralization of enamel. Knowing how this bacterium contributes to the pathophysiology of DC emphasizes how crucial it is to preserve oral health in order to stop tooth decay from getting worse [11].

Traditional and emerging prevention strategies

The main goals of traditional oral health techniques are to lessen acid production and bacterial colonization, which are the main causes of periodontal disorders and tooth caries. A key component of these strategies is the mechanical removal of plaque by brushing and flossing, which inhibits the production of biofilms and stops the growth of germs. By encouraging remineralization and preventing bacterial metabolism, chemical treatments like fluoride are essential for improving enamel resilience [12]. Regular patient adherence is necessary for these techniques to be successful, even if they are highly advised and work well when applied regularly. However, the overall efficacy of these preventative measures may be impacted by the wide variations in compliance caused by variables including age, educational attainment, availability to dental care, and personal motivation (Table 2) [13]. Inequities in oral health can also be exacerbated by socioeconomic differences that restrict access to dental treatment and goods.

**Table 2:** Preventive methods, mechanisms, and limitations of traditional dental caries management techniques.

Prevention method	Mechanism	Limitations
Fluoride therapy	Enhances enamel remineralization	Requires frequent application
Dental sealants	Creates a protective barrier	Expensive and needs professional application
Dietary modifications	Reduces sugar intake	Difficult to enforce consistently
Probiotics	Alters oral microbiome	Limited clinical validation
Genetic engineering	Modifies *Streptococcus mutans* to reduce cariogenicity	Requires further research and regulatory approval

In light of these difficulties, there is an increasing need to investigate long-term, alternative preventative strategies that do not only depend on patient compliance. By more sustainably addressing the underlying causes of oral disorders, innovations like biomimetic materials, probiotics, and new antimicrobial peptides provide encouraging remedies. For instance, without needing patient effort, bioactive compounds that release calcium and phosphate ions can help with enamel repair [14]. In a similar vein, probiotics that add good bacteria to the oral microbiome can aid in the suppression of dangerous pathogens, lowering the risk of gum disease and cavities. Furthermore, methods based on gene therapy and nanotechnology are being researched for their capacity to alter bacterial behavior and strengthen defences. The future of preventive dentistry may provide more egalitarian and successful techniques for preserving oral health by reorienting the focus from conventional, behaviour-dependent approaches to sophisticated, self-sustaining solutions [15].

Genetically engineered vaccines: mechanisms and development

By specifically targeting *S. mutans*, the main bacterial cause of tooth decay, genetic engineering has transformed the creation of vaccinations and probiotic therapies meant to prevent DC. Researchers have looked into genetically altered vaccines that trigger an immune response against important *S. mutans *virulence proteins, such as glucosyltransferases, which are necessary for the development of biofilms and the production of acid [16]. Researchers want to create protective immunity that lessens *S. mutans* colonization in the oral cavity by using recombinant antigens or live attenuated bacterial strains. Furthermore, preclinical research has demonstrated the possibility for long-term resistance to cariogenic bacteria with DNA-based vaccinations that promote mucosal immunity. Instead of only treating the symptoms of DC, these genetic engineering advancements provide a proactive approach to prevent disease at its source (Table 3) [17-19].

**Table 3:** Types of genetic engineering with their mechanisms and examples.

Type of genetic engineering	Mechanism	Example
Live attenuated strains	Reduces acidogenicity while retaining colonization ability	BCS3-L1 strain
Recombinant antigen-based vaccines	Uses specific *S. mutans* antigens to elicit an immune response	GTFs, PAc, Antigen I/II
DNA vaccines	Introduces genetic material encoding *S. mutans *antigens	In development
Passive immunization	Uses monoclonal antibodies to neutralize S. mutans	Experimental

In addition to vaccinations, probiotic treatments that outcompete *S. mutans *or counteract its negative effects have also been developed through genetic engineering. To prevent *S. mutans* from adhering to tooth surfaces and forming cariogenic biofilms, researchers have altered beneficial bacterial strains, such as *Lactobacillus* species, to create antimicrobial peptides or enzymes that specifically target *S. mutans*. Introducing genetically altered *S. mutans* strains that are unable to produce lactic acid is another novel strategy that lessens acid-induced enamel demineralization [20]. In place of conventional fluoride treatments and mechanical plaque removal, these designed probiotic therapies offer a sustainable and perhaps self-replicating method of preventing DC. Such treatments have enormous potential to revolutionize oral healthcare and lessen the prevalence of DC worldwide, provided that genetic engineering continues to improve [21].

The BCS3-L1 strain: a case study in genetic modification

An important development in the fight against DC is the genetically modified strain of *S. mutans* known as BCS3-L1. Because of their capacity to convert carbohydrates into lactic acid, which breaks down tooth enamel, traditional *S. mutans* strains are a significant cause of dental decay. In order to eradicate the damaging acid production that causes cavities, BCS3-L1 has been engineered to colonize the oral cavity and outcompete native *S. mutans* strains [22]. Researchers intend to offer a long-term, preventative oral health solution that does not rely exclusively on fluoride treatments or mechanical plaque removal by substituting this manufactured alternative for the native strains [23].

The generation of mutacin-1140, a strong lantibiotic that selectively targets and inhibits unmodified *S. mutans *strains, is a crucial characteristic of BCS3-L1. BCS3-L1 has a competitive advantage because of its selective antibacterial function, which enables it to dominate oral biofilms without endangering beneficial oral bacteria. Mutacin-1140 offers a more focused approach than broad-spectrum antibiotics, which have the potential to upset the equilibrium of the oral microbiota and cause secondary infections [24,25]. In order to preserve a healthy oral environment and stop dangerous *S. mutans* strains from recolonizing, this specificity is essential. BCS3-L1 is a novel tool for managing oral health since it employs such a tailored antibacterial approach [22].

Beyond preventing cavities, BCS3-L1 may have further advantages. This strain may lessen problems associated with oral acidity, such as dentin erosion and hypersensitivity, by decreasing the amount of acid produced in dental biofilms. Furthermore, BCS3-L1 colonization in the oral cavity over time may lessen the need for regular dental procedures, which would save money for both private citizens and public health systems [11]. To guarantee its safety, efficacy, and long-term stability in a variety of populations, comprehensive clinical trials and regulatory authorization are required prior to broad use. If BCS3-L1 is effective, it might completely transform dental treatment by offering a biological, sustainable way to stop tooth decay at its source (Table 4).

**Table 4:** Features and benefits of BCS3-L1.

BCS3-L1 strain features	Benefits
No lactic acid production	Prevents enamel demineralization
Mutacin-1140 production	Inhibits unmodified *Streptococcus mutans* strains
Long-term colonization	Provides sustained protection
Affordable treatment	Estimated cost under $100 per application

An innovative method of avoiding DC brought on by *S. mutans* is provided by the BCS3-L1 strain, which is a breakthrough in dental microbiology. To inhibit *S. mutans *and lower the risk of tooth decay, traditional techniques like fluoride treatments and antimicrobial rinses must be used consistently. These therapies do not, however, permanently change the makeup of the oral microbiome, making patients susceptible to recurring infections. BCS3-L1 is a genetically altered bacterial strain that, on the other hand, is intended to outcompete *S. mutans *and create a stable, non-cariogenic microbial habitat [26]. After a single application, BCS3-L1 offers a lifetime preventive effect by colonizing the oral cavity and displacing harmful bacteria, obviating the need for recurring treatments.

A single injection can result in the steady replacement of *S. mutans *with a harmless bacterial population, according to research on BCS3-L1 in animal models. By occupying the same ecological niche as *S. mutans*, BCS3-L1 prevents its recolonization through a process known as competitive exclusion [22]. BCS3-L1 creates a persistent presence in the oral microbiome, providing long-term protection against DC, in contrast to traditional probiotics that need to be taken on a regular basis [24]. Furthermore, research shows that the strain improves oral health by supporting a balanced microbial environment rather than impairing total microbial diversity. This strategy fits in with the increasing interest in microbiome-based treatments, which try to use good bacteria to prevent illness [16].

Beyond affecting a person's oral health, BCS3-L1 may lessen the prevalence of DC, one of the most common infectious illnesses in the world. BCS3-L1 may considerably reduce the need for restorative dental treatments and the medical expenses related to cavity treatment by offering a one-time, permanent solution [27]. In addition, its use in public health campaigns may increase access to preventive care in underprivileged areas where fluoride treatments and routine dental checkups may be scarce. However, further clinical research is required to verify its long-term safety, effectiveness, and regulatory approval prior to broad use. By changing the focus from ongoing preventative care to a single, long-lasting intervention, BCS3-L1 has the potential to completely transform dental care if it is properly adopted [2].

Safety and ethical considerations

The use of genetically modified bacterial strains, despite their promise, presents a number of issues, mostly related to biosafety, environmental effects, and ethical considerations. One significant concern is the possibility of unforeseen outcomes, such as horizontal gene transfer, in which altered genes may infiltrate native microbial populations and cause ecological disruption or the rise of infections resistant to antibiotics. Furthermore, because the long-term impacts of modified bacteria are yet unknown, their introduction into the environment presents containment and control issues [26]. The possible abuse of genetic engineering in bioterrorism or unintentional harm to human health also raises ethical questions. Widespread acceptance is also hampered by public image and regulatory issues, since concerns about genetic engineering and inadequate supervision may impede biotechnology advancement. Thorough safety evaluations, open legislation, and continuous research to guarantee the appropriate use of genetically modified bacterial strains are necessary to allay these worries (Table 5) [28].

**Table 5:** Concerns and risks associated with genetically modified vaccines.

Concern	Potential risk
Oral dysbiosis	Disrupting the natural microbiome may lead to other infections
Horizontal gene transfer	Risk of genetic material spreading to other bacteria
Systemic health effects	Potential bloodstream infections and endocarditis
Public acceptance	Skepticism toward genetically modified organisms (GMOs)

Future prospects and research directions

Several important research topics need to be solved before genetically modified vaccinations are adopted as a common caries prevention technique. First and foremost, a comprehensive assessment of these vaccines' long-term safety and effectiveness is required to make sure they do not result in off-target effects or unexpected immune reactions. In addition, for efficient defence against cariogenic bacteria like Streptococcus mutans, delivery mechanisms such as mucosal vaccination must be optimized. To ensure widespread efficacy, research is also required to comprehend any differences in immunological responses across various groups. It is also necessary to investigate ethical and regulatory issues, such as public acceptability and adherence to health regulations. Finally, in order to avoid disturbances that can result in additional oral or systemic health problems, the possible influence on the oral microbiota should be investigated (Table 6).

**Table 6:** Future studies that can improve the literature for genetically modified vaccines.

Research focus	Key considerations
Longitudinal clinical trials	Assess safety, efficacy, and microbiome stability
Regulatory approvals	Establish clear guidelines for monitoring and approval
Combination therapies	Integrate with existing preventive methods for enhanced efficacy
Synthetic biology innovations	Develop bacteria with enhanced biofilm disruption properties
Public health policy	Increase education and awareness to gain acceptance

## Conclusions

A revolutionary method of preventing dental cavities is represented by genetically modified vaccinations and bacterial strains. An innovative illustration of the potential for modified* S. mutans *to offer long-term cavity prevention is the BCS3-L1 strain. However, before these technologies can be extensively used, safety issues, moral dilemmas, and legal barriers need to be resolved. Genetically modified vaccinations have the potential to transform oral healthcare and drastically lower the prevalence of DC worldwide with more study and advancements in technology.

## References

[1] Veiga N, Figueiredo R, Correia P, Lopes P, Couto P, Fernandes GV (2023). Methods of primary clinical prevention of dental caries in the adult patient: an integrative review. Healthcare (Basel).

[2] Moore D, Allen T, Birch S, Tickle M, Walsh T, Pretty IA (2021). How effective and cost-effective is water fluoridation for adults? Protocol for a 10-year retrospective cohort study. BDJ Open.

[3] Patrick DL, Lee RS, Nucci M, Grembowski D, Jolles CZ, Milgrom P (2006). Reducing oral health disparities: a focus on social and cultural determinants. BMC Oral Health.

[4] MacDougall H (2016). Dental disparities among low-income American adults: a social work perspective. Health Soc Work.

[5] Yu X, Devine DA, Vernon JJ (2024). Manipulating the diseased oral microbiome: the power of probiotics and prebiotics. J Oral Microbiol.

[6] Das H, Janakiram C, Ramanarayanan V (2023). Effectiveness of an oral health curriculum in reducing dental caries increment and improving oral hygiene behaviour among schoolchildren of Ernakulam district in Kerala, India: study protocol for a cluster randomised trial. BMJ Open.

[7] Sivapathasundharam B, Rajendran R (2005). Shafer’s textbook of oral pathology. 5th edition.

[8] Lemos JA, Palmer SR, Zeng L (2019). The biology of Streptococcus mutans. Microbiol Spectr.

[9] Koo H, Xiao J, Klein MI, Jeon JG (2010). Exopolysaccharides produced by Streptococcus mutans glucosyltransferases modulate the establishment of microcolonies within multispecies biofilms. J Bacteriol.

[10] Georgios A, Vassiliki T, Sotirios K (2015). Acidogenicity and acidurance of dental plaque and saliva sediment from adults in relation to caries activity and chlorhexidine exposure. J Oral Microbiol.

[11] Ravuru N, Reginald BA, Reddy BS, Samatha M (2023). Relationship between dental fluorosis, dental caries and salivary levels of Streptococcus mutans. J Oral Maxillofac Pathol.

[12] Sicca C, Bobbio E, Quartuccio N, Nicolò G, Cistaro A (2016). Prevention of dental caries: a review of effective treatments. J Clin Exp Dent.

[13] Baryakova TH, Pogostin BH, Langer R, McHugh KJ (2023). Overcoming barriers to patient adherence: the case for developing innovative drug delivery systems. Nat Rev Drug Discov.

[14] Tao J, Sun Y, Wang G, Sun J, Dong S, Ding J (2025). Advanced biomaterials for targeting mature biofilms in periodontitis therapy. Bioact Mater.

[15] Shirbhate U, Bajaj P, Chandak M, Jaiswal P, Sarangi S, Suchak D, Bharti L (2023). Clinical implications of probiotics in oral and periodontal health: a comprehensive review. Cureus.

[16] Hillman JD (2002). Genetically modified Streptococcus mutans for the prevention of dental caries. Antonie Van Leeuwenhoek.

[17] Huang Y, Hajishengallis G, Michalek SM (2001). Induction of protective immunity against Streptococcus mutans colonization after mucosal immunization with attenuated Salmonella enterica serovar typhimurium expressing an S. mutans adhesin under the control of in vivo-inducible nirB promoter. Infect Immun.

[18] de Souza Pereira G, Batista MT, Dos Santos NF (2022). Streptococcus mutans glutamate binding protein (GlnH) as antigen target for a mucosal anti-caries vaccine. Braz J Microbiol.

[19] Smith DJ (2002). Dental caries vaccines: prospects and concerns. Crit Rev Oral Biol Med.

[20] Luo SC, Wei SM, Luo XT, Yang QQ, Wong KH, Cheung PC, Zhang BB (2024). How probiotics, prebiotics, synbiotics, and postbiotics prevent dental caries: an oral microbiota perspective. NPJ Biofilms Microbiomes.

[21] Zhang J, Wang Q, Duan Z (2024). Preventive effects of probiotics on dental caries in vitro and in vivo. BMC Oral Health.

[22] Hillman JD, Brooks TA, Michalek SM, Harmon CC, Snoep JL, van Der Weijden CC (2000). Construction and characterization of an effector strain of Streptococcus mutans for replacement therapy of dental caries. Infect Immun.

[23] Pradeep P, Thomas AR, Kaur K, Sarah Samson R, Mayya A, Adiga S, Kumbargere Nagraj S (2024). Herbal medicines to prevent dental caries. Cochrane Database Syst Rev.

[24] Smith L, Zachariah C, Thirumoorthy R, Rocca J, Novák J, Hillman JD, Edison AS (2003). Structure and dynamics of the lantibiotic mutacin 1140. Biochemistry.

[25] Kers JA, Sharp RE, Defusco AW (2018). Mutacin 1140 lantibiotic variants are efficacious against Clostridium difficile infection. Front Microbiol.

[26] Frey J (2007). Biological safety concepts of genetically modified live bacterial vaccines. Vaccine.

[27] Giacaman RA, Fernández CE, Muñoz-Sandoval C (2022). Understanding dental caries as a non-communicable and behavioral disease: management implications. Front Oral Health.

[28] Bohua L, Yuexin W, Yakun O, Kunlan Z, Huan L, Ruipeng L (2023). Ethical framework on risk governance of synthetic biology. J Biosaf Biosecu.

